# Comparison of Three Automated Measurements of Ventricular Volumes of the Brain

**DOI:** 10.1007/s10278-025-01819-6

**Published:** 2026-01-20

**Authors:** Xue Zhang, Yonggang Li, Ping Mu, Xiaotong Yu, Xiu Zhang, Shulin Liu, Baishi Wang, Ning Li, Fu Ren

**Affiliations:** 1 Department of Human Anatomy, Shenyang Medical College, Shenyang, Liaoning 110034 People’s Republic of China; 2 Liaoning Province Key Laboratory for Phenomics of Human Ethnic Specificity and Critical Illness (LPKL-PHESCI), Shenyang, Liaoning 110034 People’s Republic of China; 3 Shenyang Key Laboratory for Phenomics, Shenyang, Liaoning 110034 People’s Republic of China; 4 Department of Physiology, Shenyang Medical College, Shenyang, Liaoning 110034 People’s Republic of China; 5 Department of Stomatology, Shenyang Medical College, Shenyang, Liaoning 110034 People’s Republic of China; 6 Harbin Medical University, Daqing, Heilongjiang 163711 People’s Republic of China; 7 Department of Biochemistry and Molecular Biology, Shenyang Medical College, Shenyang, Liaoning 110034 People’s Republic of China

**Keywords:** Ventricular volumetry, Automated segmentation, Open-source software, Method validation, Neuroimaging

## Abstract

Accurate segmentation and measurement of ventricular volume are critical for neuroscience research and neurological disease diagnosis. In resource-limited settings, free and open-source automated tools offer accessible solutions. However, the lack of comparative evaluations limits their application. This study aims to identify reliable free tools for automated ventricular volume measurement to support clinical and research utilization. Magnetic resonance imaging (MRI) data from 150 healthy adults were collected with informed consent. Ventricular volumes were segmented using three open-source tools (3D Slicer, FreeSurfer, ITK-SNAP) and compared with manual segmentation as the reference standard. Pearson’s correlation coefficient, intraclass correlation coefficient (ICC), and the Bland–Altman analysis were employed to evaluate consistency and reliability. All three automated tools showed significant correlations with manual measurements (*P* < 0.01). ITK-SNAP had the highest Pearson correlation and ICC values, followed by 3D Slicer, while FreeSurfer had the lowest. All tools demonstrated strong reliability, with ICCs greater than 0.9. The Bland–Altman analysis showed that ITK-SNAP had the closest consistency with manual results, again followed by 3D Slicer, with FreeSurfer performing least consistently. ITK-SNAP demonstrates higher accuracy and reliability for ventricular volumetry compared to 3D Slicer and FreeSurfer. Its open-source nature supports broader implementation in resource-constrained environments, enhancing neuroimaging accessibility for clinical and research applications.

**Supplementary Information:**

The online version contains supplementary material available at https://doi.org/10.1007/s10278-025-01819-6.

## Introduction

In recent years, continuous advances in magnetic resonance imaging (MRI) have made the technique indispensable to brain-science research, particularly for quantitative analyses of brain structure. MRI not only depicts fine neuroanatomy but also reveals pathological changes linked to a wide range of neurological and psychiatric disorders [1–3]. Among the many structural metrics derived from MRI, ventricular volume is especially important. The brain’s ventricles are cerebrospinal-fluid-filled cavities, and changes in their volume serve as key diagnostic indicators of neurological disease [4]. Recently, numerous studies have documented ventricular enlargement in Parkinson’s disease [5–7], Alzheimer’s disease [8–10], schizophrenia [11, 12], and related conditions. Although some work has examined sex differences in ventricular size [13, 14], precise, age-stratified reference values remain scarce—largely because most studies have not standardized ventricular-volume data. Accurate ventricular-volume measurement is therefore critical for neuroscience research, clinical diagnosis, disease monitoring, and even anthropological studies. For example, early Alzheimer’s disease is characterized by neuronal loss and atrophy of the hippocampus and temporal lobes [15], leading to ventricular expansion. In normal-pressure hydrocephalus [16], enlarged ventricles occur despite normal intracranial pressure and typically present with gait disturbance, cognitive decline, and urinary incontinence; precise volume assessment helps predict current and future motor and cognitive changes [17, 18]. Dynamic tracking of ventricular size also provides a visual index of disease progression. In late-stage Parkinson’s disease, brain atrophy can enlarge the ventricles; regular measurements help gauge progression rate and prognosis [19]. After traumatic brain injury, rapid ventricular enlargement may signal post-traumatic hydrocephalus [20], where frequent ventricular volume checks can detect neurological deterioration early and mitigate treatment delays. Finally, ventricular volume serves as an objective metric of therapeutic efficacy. Serial measurements after shunt placement or neuroendoscopic surgery for hydrocephalus reveal how effectively the procedure relieves cerebrospinal-fluid obstruction. In brain-tumor patients undergoing chemotherapy or targeted therapy, tracking ventricular size can show whether tumor shrinkage improves ventricular pressure, guiding subsequent treatment decisions. In short, high-precision ventricular-volume assessment—integrated with clinical findings and ancillary tests—enables early disease detection, timely intervention, and optimized long-term management [15].

Since ventricular size cannot be assessed accurately through simple visual inspection [14], researchers have developed several quantitative techniques. One common approach is the use of linear indices, in which the ventricular width is measured on selected tomographic slices. This method is quick, reliable, and reproducible, making it suitable for routine neurosurgical practice [21]. However, linear measurements still require manual input, can be time-consuming, and are subject to observer bias. Manual planimetry remains the gold standard for ventricular-volume quantification [22, 23]. In this method, trained specialists trace the ventricular boundaries on each MRI slice, calculate the cross-sectional area, multiply by slice thickness, and sum the results to obtain total volume. Although highly accurate, the process is labor-intensive and thus practical only for small-sample studies; it is ill-suited to the large datasets encountered in modern research and clinical practice. Moreover, the technique demands substantial anatomical expertise, and inter-observer variability can introduce systematic discrepancies [22, 24, 25].

Deep-learning frameworks—particularly convolutional neural networks (CNNs) [14, 25, 26] and other machine-learning approaches [27, 28]—have recently enabled fully automated, high-precision segmentation and quantification of intracranial structures. Still, their performance hinges on large, diverse, well-labelled training datasets, consistent acquisition protocols, and ample computational resources; shortcomings in any of these areas can degrade accuracy. Automated volumetric tools offer an attractive alternative. By exploiting tissue-density differences directly in the raw MR data, they compute brain-structure volumes without a priori region-of-interest definitions or programmer intervention. This automation eliminates observer bias, minimizes human error, and delivers measurements that are objective, reproducible, and scalable—qualities essential for reliable diagnosis and longitudinal monitoring.

Several free, open-source packages—FreeSurfer [29, 30], SPM [31], MRI Studio [22], 3D Slicer [32, 33], and ITK-SNAP [33]—already provide ventricular-volume segmentation and calculation routines [34–36]. Comparative studies confirm their value: Keshavan et al. [37] reported a very high correlation between point-count stereotaxy and an algorithmic MRI-based method for lateral-ventricular volume in 18 subjects. Ertekin et al. [38] showed that both ALVIN (automated) and manual ImageJ tracing yield reliable lateral-ventricular and intracranial volumes in Alzheimer’s patients and age-matched controls. Acer et al. [39] found no significant differences—and strong agreement—between Cavalieri point-counting and planimetry for volumes of the lateral, third, and fourth ventricles.

Nevertheless, segmentation performance can vary across tools and ventricular subregions. Small, non-continuous recesses (e.g., anterior, posterior, and inferior horns) are particularly prone to misidentification, given the ventricular system’s intricate geometry [40]. Although free software lowers financial barriers, the scarcity of head-to-head evaluations limits widespread adoption in clinical and research settings.

In this study, we measured volumes of the lateral, third, and fourth ventricles—as well as total ventricular volume—in healthy Han Chinese adults to establish normative reference ranges. Manual planimetry with ImageJ served as the reference standard. We then assessed three widely used, open-source platforms—3D Slicer, FreeSurfer, and ITK-SNAP—examining their correlation, reliability, and consistency with manual measurements. By clarifying the strengths and limitations of each tool, our goal is to simplify access to accurate, cost-free ventricular volume analysis for researchers and clinicians, thereby providing a robust methodological foundation for diagnosing and monitoring ventricle-related disorders.

## Materials and Methods

### Subjects

Participants were recruited using convenience sampling among healthy volunteers meeting strict inclusion criteria. This non-random sampling approach is acknowledged as a limitation. A cohort of 150 eligible participants (60 males; 90 females) aged 18–30 years was systematically recruited from healthy populations through rigorous implementation of predefined selection criteria to ensure sample representativeness and scientific validity. Inclusion criteria comprised: (1) Han Chinese ancestry with ≥ 3 generations of permanent residency in Liaoning Province, China, and native Mandarin speakers; (2) Absence of systemic disorders, neurological conditions, cranial trauma, or psychiatric history. Exclusion criteria included: (1) Current psychiatric diagnoses or psychological abnormalities; (2) Severe hepatic/renal insufficiency or major organ dysfunction; (3) Implanted medical devices (pacemakers, metallic stents, or orthopedic fixation plates). Anthropometric parameters (height/weight) were documented to control for potential confounding effects of extreme obesity or growth retardation. All participants provided written informed consent prior to study enrollment.

### Experimental Data

#### Data Acquisition

MRI data were acquired using a 1.5 T Siemens Sonata scanner (Siemens Medical Solutions, Germany). All participants were positioned in the standard oculo-auricular plane with the following scan orientations: axial slices parallel to the canthomeatal line, coronal slices perpendicular to the canthomeatal line, and sagittal slices parallel to the falx cerebri. Specific scanning parameters are as follows: repetition time, 9.2 ms; echo time, 3.6 ms; inversion time, 350 ms; flip angle, 13°; field of view, 256 × 256 mm; matrix size, 512 × 512 × 192 mm; 192 slices; slice thickness, 1 mm; voxel dimensions, 1 × 1 × 1 mm. Whole-brain coverage was obtained to ensure comprehensive neuroanatomical data acquisition. Prior to scanning, participants received standardized instructions to maintain head stillness, thereby minimizing motion artifacts. The imaging protocol employed a T1-weighted three-dimensional brain volume (3D-BRAVO) sequence. The parameters were selected to maximize spatial resolution and signal-to-noise ratio while ensuring compatibility with subsequent image processing pipelines. All images were stored in DICOM format for further analysis.

#### Data Screening and Pre-processing

Rigorous quality screening is essential when processing MRI data. This step combines automated and manual procedures—including artefact detection and head-motion correction—to ensure that only high-quality images proceed to analysis. Raw volumes were inspected in MRIcroGL (https://www.nitrc.org/projects/mricrogl), which efficiently discards scans with excessive artefacts or incomplete coverage, thereby safeguarding the reliability of downstream analyses. If a participant’s scan fails quality control in any one pipeline, that participant’s data are removed from all pipelines to maintain a consistent sample size across measures. Ventricular segmentation was performed in 3D Slicer and ITK-SNAP: signal-intensity thresholds were set manually, then refined with semi-automatic algorithms to exclude non-compliant images.

In FreeSurfer, each volume was first registered to the Talairach atlas and corrected for the B1 bias field. The resulting normalized, high-resolution T1-weighted images were skull-stripped to remove extracerebral tissue, after which the cortex, subcortical white matter, and deep grey-matter structures were segmented by defining grey/white-matter boundaries [41]. Volumes that failed FreeSurfer’s built-in quality checks were excluded.

Finally, of 150 initially enrolled participants, 40 scans were excluded during quality screening, leaving 110 datasets for final analysis.

**Fig. 1:**
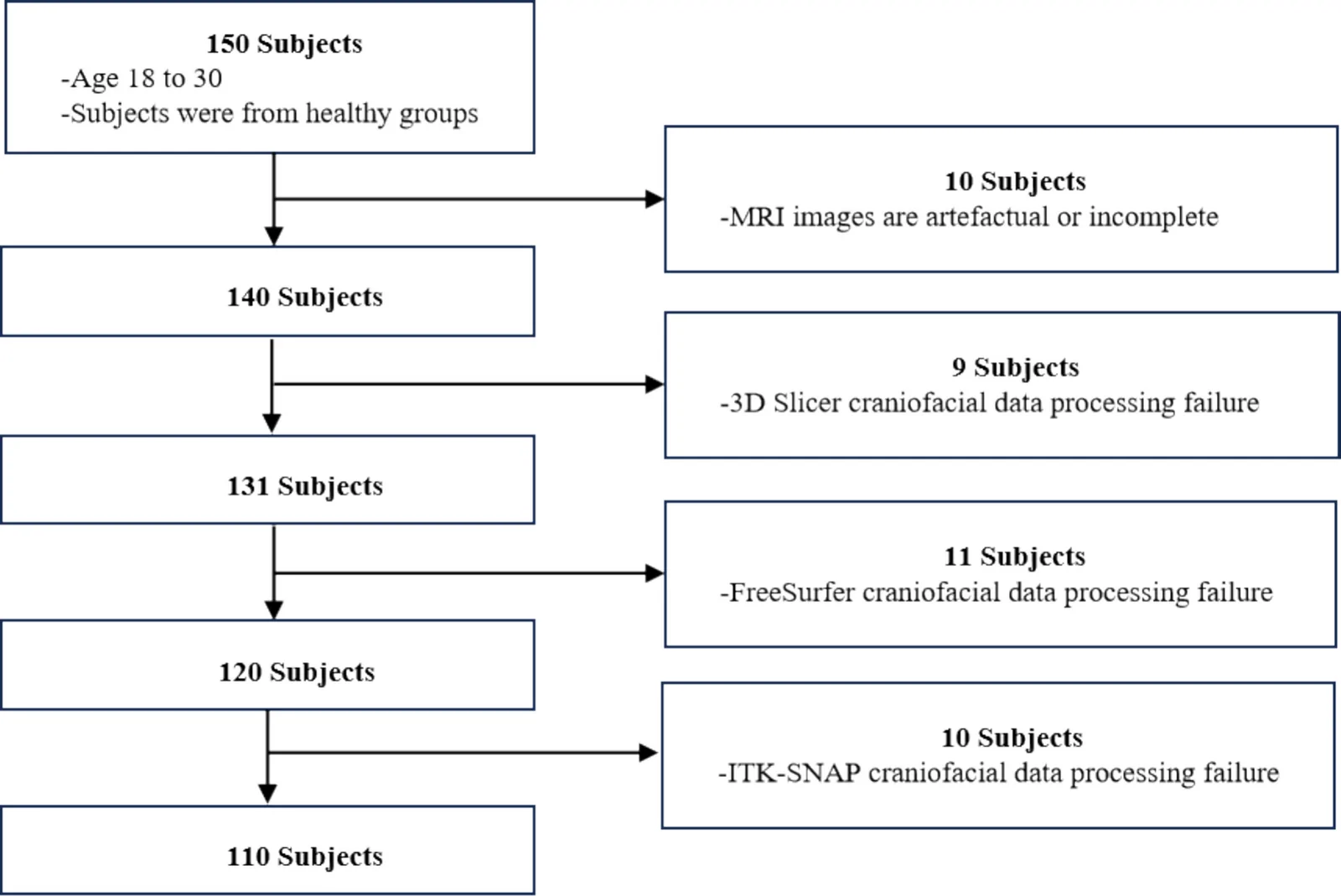
Flowchart of MRI data screening of subjects

Figure 1 illustrates the MRI screening workflow, and Table 1 summarizes the characteristics of the final sample.

**Table 1:** Demographics of subject population

Gender	Number of subjects (percentage of total)	Average age (years) ± standard deviation
Male	39 (35.5%)	24.13 ± 2.811
Female	71 (64.5%)	24.74 ± 2.254

### Image Processing and Volume Measurement

#### Manual Measurement

The ventricular and cranial cavity volumes were manually measured using ImageJ software (https://imagej.net). Manual segmentation was conducted by one trained neuroscientist and reviewed by a neuroradiologist to ensure anatomical accuracy. Intra-rater reliability was evaluated by repeating manual measurements for 20 randomly selected subjects after a 2-week interval, yielding an ICC of 0.961 (95% CI 0.942–0.975). The operator performing manual segmentation was blinded to automated outputs.

The methodology involves the following steps:

Image segmentation: A threshold is established based on grayscale intensity differences within the image, allowing segmentation of pixels into distinct regions. Regions of interest (ROIs) are then identified and manually outlined.

Area calculation: The cross-sectional area of each ROI is quantified.

Volume estimation: Total volume is computed by multiplying the cumulative cross-sectional surface area by the cross-sectional thickness.

For cranial cavity volume, the product of three orthogonal diameters was utilized:

Transverse (left–right) diameter: Measured as the maximum horizontal distance between the left and right cranial bones, intersecting the midpoint of the midbrain duct.

Superior–inferior (vertical) diameter: Defined as the maximum vertical distance in the median-sagittal plane, extending from the foramen magnum of the occipital bone to the parietal bone.

Anteroposterior (front-back) diameter: Recorded as the maximal anterior–posterior distance in the median sagittal plane, passing through the inferior margin of the corpus callosum.

**Fig. 2:**
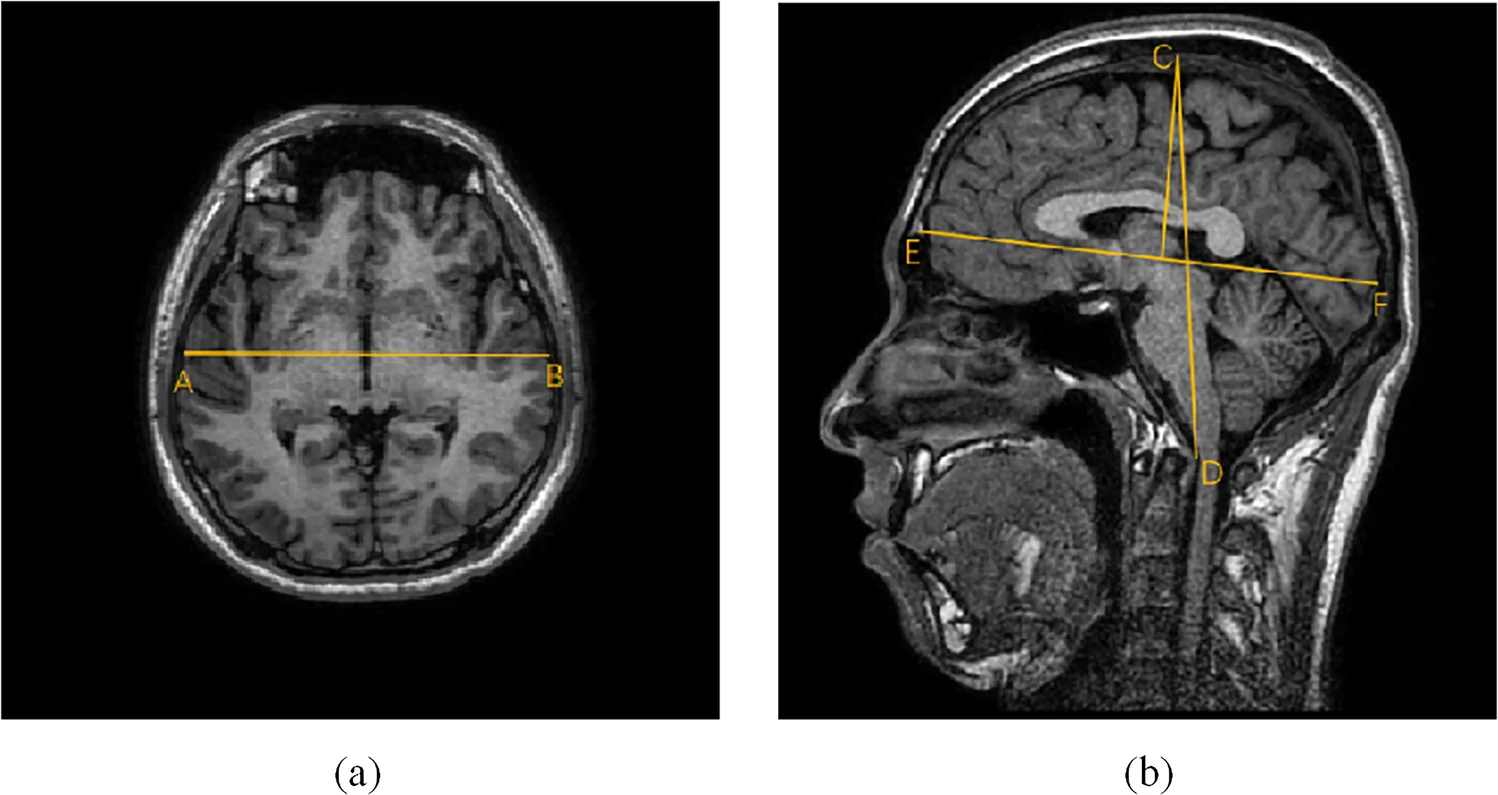
Calculation of the volume of the cranial cavity by measuring the right and left diameters of the cranial cavity, the anterior and posterior diameters, and the upper and lower diameters. Where the cranial cavity right and left diameters AB were measured in the horizontal plane (**a**) and the cranial cavity upper and lower diameters CD and cranial cavity anterior and posterior diameters EF were measured in the sagittal plane (**b**)

All diameter measurements were repeated three times, and the mean value was used for final calculations. The schematic representation of these measurements is provided in Fig. 2.

#### Ventricular Volume Measurement Protocol

The ventricular volume measurement was performed using ImageJ software with the following workflow:

1. Data import and initial setup
  Cranial MRI scans of study subjects were imported into ImageJ.
  The “Use virtual stack” option was selected to open sequential images.
  Image scale was calibrated (Analyze → Set Scale) with units set to millimeters (mm).
2. Boundary delineation
  The Magic Wand tool (threshold-adjusted) was used to automatically detect ventricular boundaries.
  Manual refinement was applied to outline the lateral ventricles, third ventricle, and fourth ventricle across all slices (Fig. 3).
  **Fig. 3:**
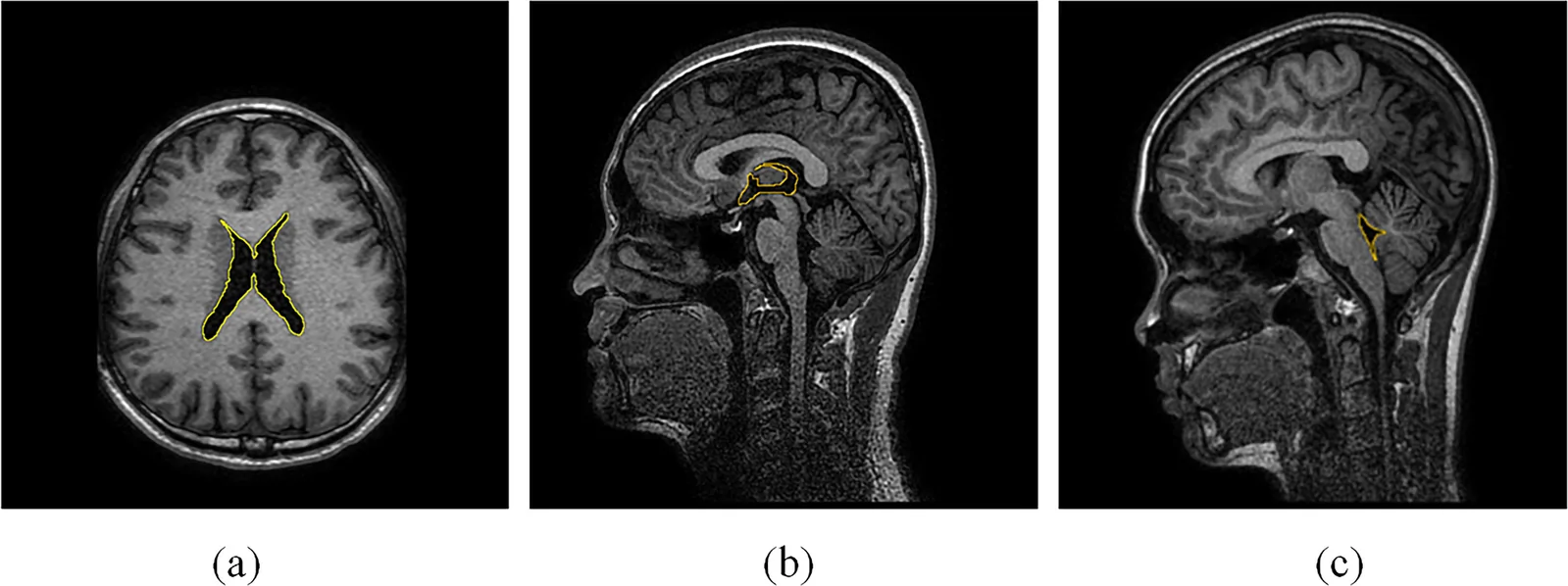
Schematic diagram of the ventricles outlined using the manual measurement tool ImageJ, with the lateral ventricles (**a**) outlined in the horizontal plane and the third (**b**) and fourth (**c**) ventricles outlined in the sagittal plane
3. ROI management and area calculation
  Outlined regions were added to the ROI Manager (Analyze → Tools → ROI Manager → Add).
  Cross-sectional areas (mm^2^) were measured (Analyze → Set Measurements → Area), and data were exported to Excel for summation.
4. Volume computation
  Total ventricular volume (mm^3^) was calculated as:
  $$Volume\:=\:\sum(Layer\;Surface\;Area)\:\times\:Section\;Thickness$$
  The protocol was repeated for all 110 subjects to measure volumes of all ventricular structures.

## Automated Measurement

This study employed three automated measurement software packages—3D Slicer, FreeSurfer, and ITK-SNAP—to quantify the volumes of the lateral ventricles, third ventricle, fourth ventricle, and ventricular system. 3D Slicer version 4.11 and ITK-SNAP version 4.0.1 were run on the same Windows System. FreeSurfer version 7.4.1 was run on a Linux Workstation. 3D Slicer, FreeSurfer, and ITK-SNAP are freely available at https://www.slicer.org, https://surfer.nmr.mgh.harvard.edu, and http://www.itksnap.org. ITK-SNAP and 3D Slicer required moderate user interaction for parameter tuning and boundary refinement, while FreeSurfer was fully automated.

Below is the detailed methodology for 3D Slicer:

1. Overview of 3D Slicer‌
  3D Slicer is a free, open-source platform for medical image analysis, offering tools for:
  Interactive segmentation.
  Visualization (including volume rendering).
2. Segmentation workflow
  1) Image import and preprocessing.
   DICOM-formatted cranial MRI data were loaded (Load DICOM Data → Load).
   The 3D viewer was adjusted for optimal tissue contrast (window width/level).
  2) Axial plane annotation
  Primary segmentation was performed in the axial plane to delineate ventricular boundaries (Modules → Segment Editor → Add Level Tracing).
  ROIs were labeled layer–by–layer (Fig. 4).
  **Fig. 4:**
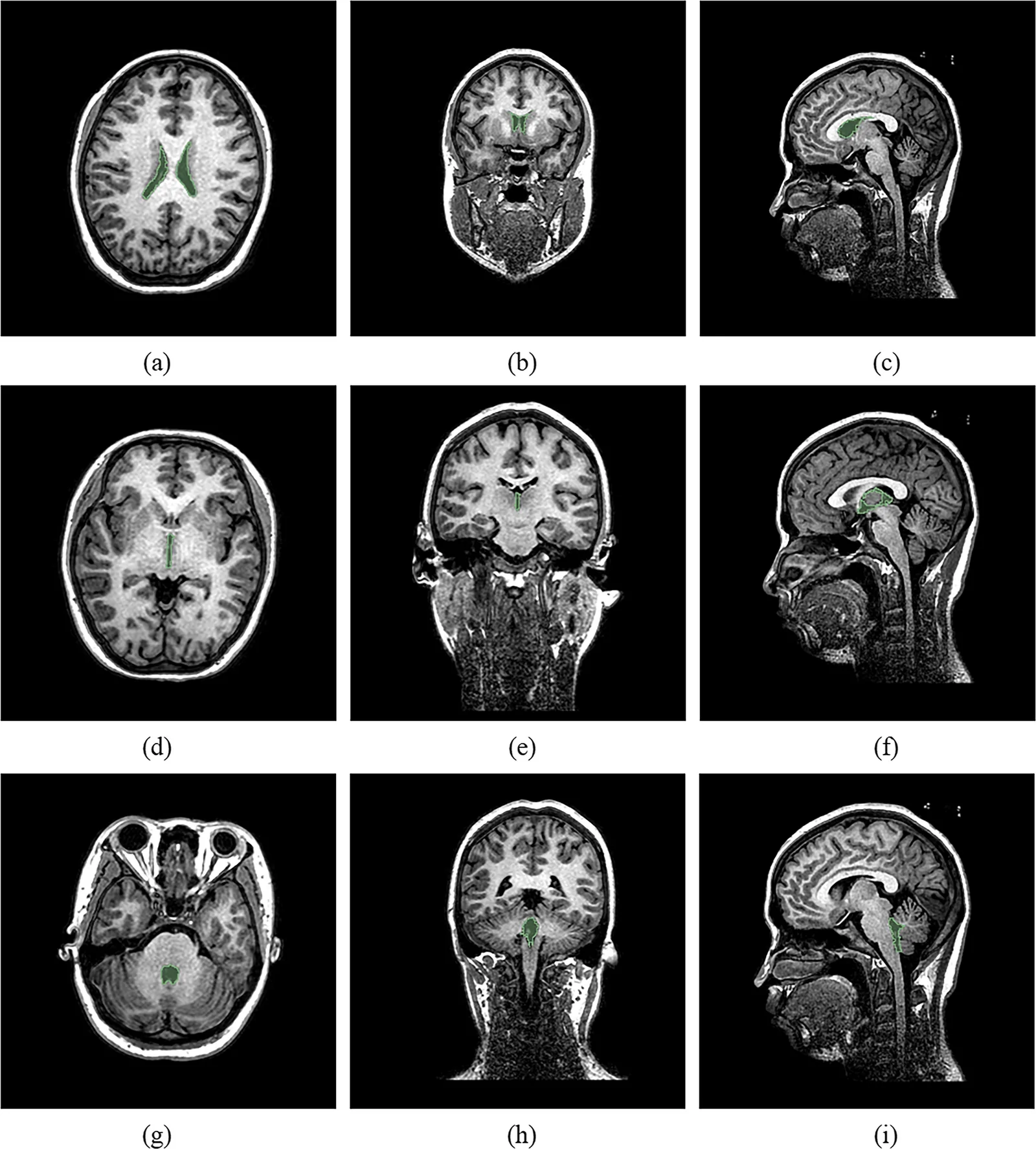
3D Slicer segmentation of each ventricle. Green color shows the segmentation region of each ventricle, **a**–**c** the segmentation region of lateral ventricle in axial, coronal, and sagittal positions, respectively; **d**–**f** the segmentation region of the third ventricle in axial, coronal, and sagittal positions, respectively; **g**–**i** the segmentation region of the fourth ventricle in axial, coronal, and sagittal positions, respectively
  3) Multi-planar verification
   Sagittal and coronal planes were reviewed for completeness.
   Errors were corrected using Erase and Paint tools (based on signal intensity).
  4) 3D reconstruction and volume calculation
    Show 3D-generated volumetric renderings.
   Ventricular volumes (mm^3^) were computed automatically (Quantification → Segment Statistics → Scalar Volume).
3. Repetition and scalability
  The protocol was replicated for all 110 subjects to ensure consistent measurements across the cohort.
  FreeSurfer software can be used to analyze and visualize brain anatomical structures and functional imaging data. To extract reliable volume values, images are automatically processed in FreeSurfer using longitudinal flow [42]. Specifically, unbiased within-subject template spaces and images are created using robust, inverse-consistent alignment [43]. The generic information of the within-subject templates is then used to initialize several processing steps, such as cranial stripping, Talairach transform, atlas alignment, and spherical mapping and wrapping, significantly improving reliability and statistical power [43].
  All subjects underwent fully automated Free Surfer “recon-all” pipeline processing. Use the script “recon-all–ifile.dcm–subjectbert–all” for fully automatic reconstruction. The specific steps are as follows:
  (1) Configure the FreeSurfer environment:
  FREESURFER_HOME/home/user/freesurfer.
  FSFAST_HOME/home/user/freesurfer/fsfast.
  FSF_OUTPUT_FORMAT nii.gz.
  SUBJECTS_DIR/home/user/freesurfer/subjects.
  MNI_DIR/home/user/freesurfer/mni.
  (2) Enter the destination folder: cd/home/user/free surfer/subjects.
  (3) Convert MRI data format: The.dcm format files of the preprocessed MRI data were converted to.nii.gz format by MRIcroGL software, and subsequently imported into subjects named MALE01, MALE02, MALE03… where the cranial 3D images were named male1/1_male, male2/2_male, male3/3_male…. male, male2/2_male, male3/3_male…
  (4) Execute the recon-all command: recon-all -s MALE01 -i male1/1_male.nii.gz -all.
  (5) Extraction of subcortical volume data: asegstats2table –subjects MALE1 –meas volume –tablefile MALE1volume.txt.
  (6) Export data: Open the data in the.txt file and import it into Excel sheet to get the volume of all subcortical structures, including the lateral ventricle, the third ventricle, the fourth ventricle, and the volume of the ventricular system, and the unit of the volume of all ventricles is mm^3^.
  (7) Visualization of results: enter Freeview and open the corresponding volume data folder to display the tomographic segmentation images (see Fig. 5) and 3D visualization images for each face.
  **Fig. 5:**
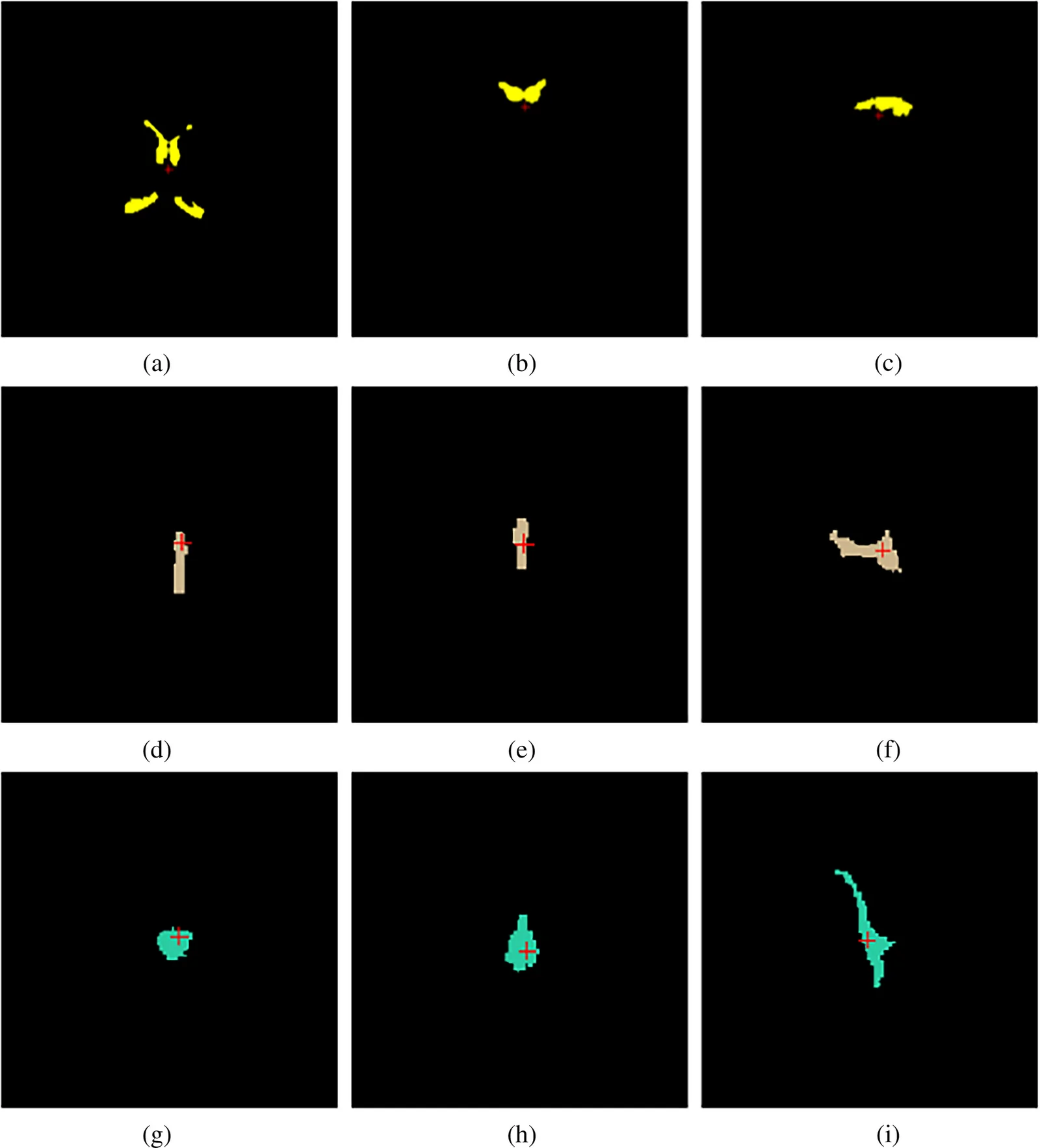
FreeSurfer segmentation maps of each ventricle. **a**–**c** Lateral ventricular segmentation regions in axial, coronal, and sagittal positions, respectively (yellow); **d**–**f** third ventricular segmentation regions in axial, coronal, and sagittal positions, respectively (brown); **g**–**i** fourth ventricular segmentation regions in axial, coronal, and sagittal positions, respectively (green)
  ITK-SNAP uses an active contour model to segment images. This model evolves a contour to fit tightly to the target boundary in the image by defining an energy minimization problem [44]. The basic principle is that the user can guide the segmentation process by placing seed points on the image, and these seeds automatically evolve into a closed contour based on the image features and user-defined parameters. Starting from one or more seed points, neighboring pixels are gradually merged into the region where the seed points are located according to a predefined similarity criterion. In ventricular volume measurements, one or more seed points, which are usually located within the ventricle, are first manually selected in the MRI image. Then, the surrounding pixels are gradually merged into the ventricles based on similarity criteria such as grey value and texture features. Finally, the number of pixels within the ventricle is counted and the volume of the ventricle is calculated [23].
  The specific measurement method is as follows: first, open the subject’s cranial MRI data, import the cranial MRI images into the software; then, select the region of interest, i.e., each ventricle, select “Segment 3D,” set “Thresholding” so that the ventricles and surrounding tissues appear with obvious boundaries; next, segment the ROI, add seed points, adjust the parameters, and try to make them evenly distributed throughout the segmentation region, click “Enter” to enter the segmentation region. Then, select the region of interest, i.e., each ventricle, choose “Segment 3D,” set “Thresholding” to make the ventricles and the surrounding tissues appear with obvious boundaries; next, segment the ROI, add the seed points, adjust the parameters, try to make them evenly distributed in the whole segmentation region, click “Enter” to enter the automatic segmentation, and click “Stop” to stop the segmentation when it is completed. When adjusting the parameters, the changes in the segmentation results can be displayed in real time to provide immediate feedback; modification and adjustment: return to the main interface to check whether the automatic segmentation of the ROI is complete, and then manually modify and adjust the ROI; after that, carry out the three-dimensional reconstruction, return to the main interface to present the segmentation of the ventricles in various directions, see Fig. 6, and select “3D” to present the three-dimensional reconstruction. Choose “3D” to present the 3D visualization image; calculate the volume: choose “segmentation-volumes and statistics” to automatically calculate the volume of each ventricle. Finally, the above steps were repeated to measure the volumes of the lateral ventricles, the third ventricle, the fourth ventricle, and the ventricular system in 110 subjects.
  **Fig. 6:**
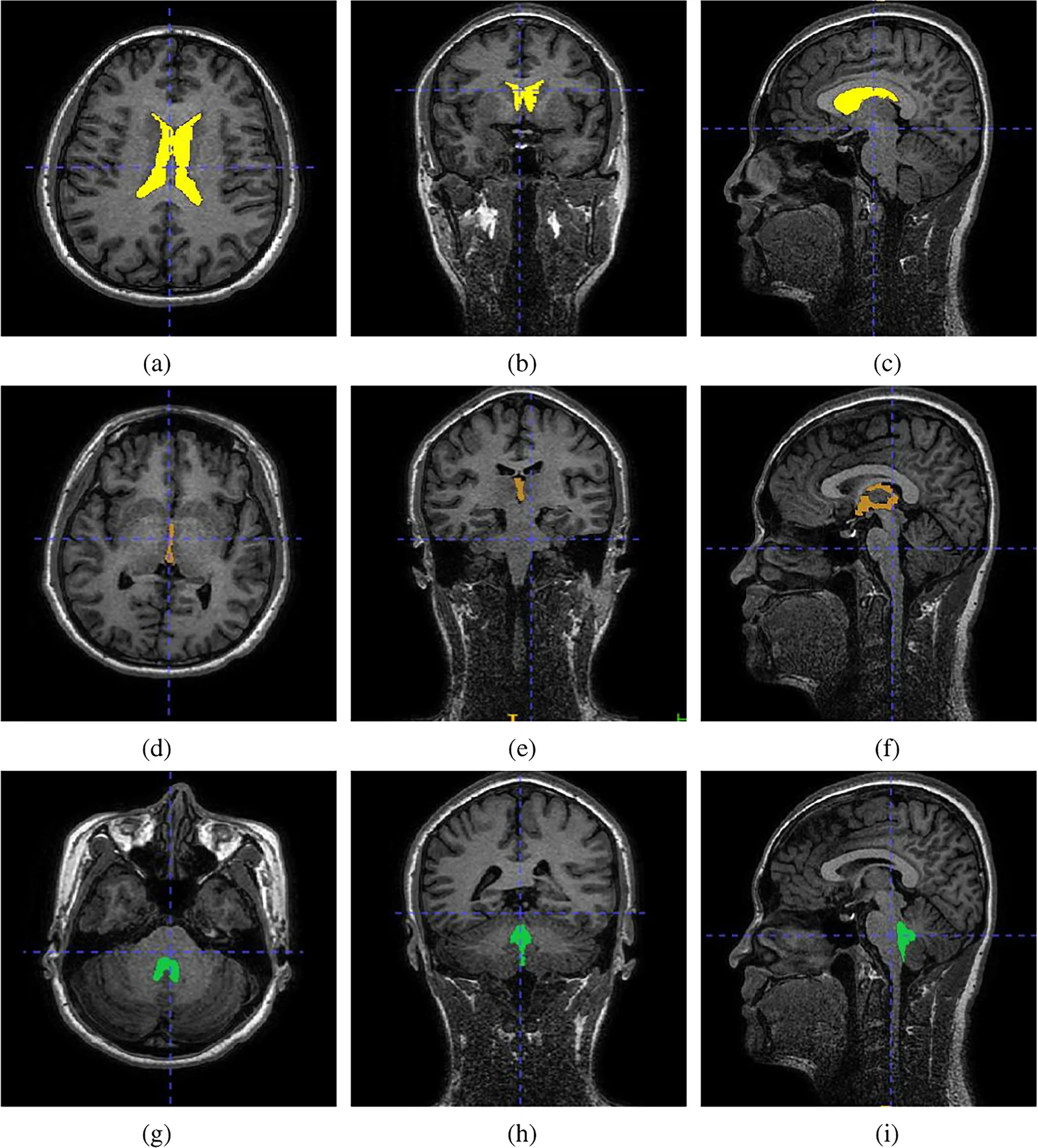
ITK-SNAP segmentation maps of each ventricle. **a**–**c** Lateral ventricular segmentation regions in axial, coronal, and sagittal positions, respectively (yellow); **d**–**f** third ventricular segmentation regions in axial, coronal, and sagittal positions, respectively (brown); **g**–**i** fourth ventricular segmentation regions in axial, coronal, and sagittal positions, respectively (green)

### Data Processing

To evaluate the accuracy and consistency of the three methods, their measurements were compared against manual measurements, which served as the reference standard. For the manual method, both ventricular volume and each cranial cavity diameter were measured three times, and the average was used for analysis. To eliminate the influence of individual differences in head size on the measurement results, all raw data were standardized using the following formula:

Standardized ventricular volume = (Original ventricular volume/Cranial cavity volume) × Mean cranial cavity volume.

### Statistical Methods

The ventricular volumes measured using the four measurement methods in this study were quantitative and described using mean ± standard deviation $\overline {X}\mathit\pm\mathrm{S}$. The results of the different measurement methods were statistically tested in SPSS 25.0 and Origin Pro using the following method, and a difference of *P* < 0.05 indicated statistical significance. Independent samples *t*-tests were used to compare whether there were differences in ventricular volumes between the sexes. Pearson’s correlation coefficient was used to assess the correlation between the ventricular volumes measured by the manual method and the three automated methods. The value of the correlation coefficient *r* ranged between − 1 and 1, with *r* < 0.2 indicating that the two variables were essentially uncorrelated, between 0.2 and 0.4 indicating a low correlation, between 0.4 and 0.6 indicating a moderate correlation, between 0.6 and 0.8 indicating a strong correlation, and correlation coefficients between 0.8 and 1.0 indicating a very strong correlation. The reliability of the three automated methods for measuring ventricular volumes was assessed using ICC. Values of ICC ranged from 0 to 1, with 0 indicating unreliable and 1 indicating completely reliable, and values of ICC lower than 0.5 indicating poor reliability, between 0.5 and 0.75 indicating moderate reliability, between 0.75 and 0.9 indicating good reliability, and greater than 0.9 indicating excellent reliability. The Bland–Altman analysis performed in OriginPro 2024 (https://www.originlab.com), which was used to assess the agreement between the results of the different automated measurement methods and the results of the manual measurements. This method assesses the consistency and interchangeability between different methods by plotting scatter plots of the difference of measurement results against the mean and analyzing the systematic and random errors in the measurement results of different methods. The upper and lower dashed lines in the results plot of the Bland–Altman analysis represent the upper and lower limits of the 95% LoA, and the long solid line in the middle represents the mean of the differences. The 95% limit of agreement is an important indicator for assessing the agreement between different methods. This range covers 95% of the difference, and if 95% of the difference lies within this limit, the two methods are considered to be in good agreement. The narrower the LoA, the higher the degree of agreement between the two methods. Through the above analysis steps, this study will comprehensively assess the relevance, reliability, and consistency of the three automatic measurement methods for ventricular volume measurement, lay a solid foundation for the development of large-sample studies on ventricular volume, provide a scientific basis for clinical application, and help clinicians to quickly and accurately assess the ventricular condition.

## Results

### Measurement of Ventricular Volume Values by Three Automated and Manual Measurement Methods

In order to test whether there is a difference between the raw data and the standardized data, an independent samples *t*-test was performed on the raw and standardized data of ventricular volumes measured by ImageJ, 3D Slicer, FreeSurfer, and ITK-SNAP, and the results are shown in Table 2. The results showed that there was no significant difference (*P* > 0.05) between the raw and standardized data of lateral ventricle volume, third ventricle volume, fourth ventricle volume, and ventricular system volume between the manual and the three automated measurement methods. Volume and ventricular system volume were not significantly different between the raw and standardized data (*P* > 0.05). To ensure the reliability of the analysis results, standardized data were used for statistical analysis in this study.

**Table 2:** ImageJ, 3D Slicer, FreeSurfer, and ITK-SNAP results for ventricular volume original and standard values ($\overline{X }$±S)

		Original value (mm^3^)	Standard value (mm^3^)	*T*	*P*-value	Mean difference	Standard deviation difference
ImageJ	LVV	14,552.68 ± 3847.64	14,540.92 ± 3707.54	0.023	0.982	11.76	509.46
	TVV	1087.51 ± 296.01	1089.68 ± 290.43	− 0.055	0.956	− 2.17	39.54
	FVV	1659.34 ± 430.43	1657.86 ± 410.43	0.026	0.979	1.49	56.71
	VV	17,299.54 ± 4171.88	17,288.47 ± 3975.32	0.020	0.984	11.07	549.44
3D Slicer	LVV	14,900.94 ± 4517.45	14,886.17 ± 4349.08	0.025	0.980	14.77	597.89
	TVV	1062.72 ± 290.26	1065.15 ± 286.86	− 0.063	0.950	− 2.44	38.91
	FVV	1655.19 ± 436.18	1653.56 ± 416.71	0.028	0.978	1.62	57.52
	VV	17,618.84 ± 4843.91	17,604.89 ± 4623.22	0.022	0.983	13.96	638.45
FreeSurfer	LVV	13,122.90 ± 4285.33	13,084.36 ± 4039.01	0.069	0.945	38.54	561.47
	TVV	1050.21 ± 260.53	1053.09 ± 257.36	− 0.082	0.934	− 2.87	34.92
	FVV	1658.29 ± 411.77	1657.12 ± 390.90	0.021	0.983	1.16	54.13
	VV	15,831.40 ± 4574.21	15,794.56 ± 4267.69	0.062	0.951	36.83	596.48
ITK-SNAP	LVV	13,844.69 ± 4194.86	13,814.01 ± 4006.95	0.055	0.956	30.68	553.11
	TVV	1057.41 ± 290.98	1059.41 ± 285.97	− 0.051	0.959	− 2.00	38.90
	FVV	1660.96 ± 433.97	1659.47 ± 414.32	0.026	0.979	1.49	57.21
	VV	16,563.06 ± 4520.46	16,532.89 ± 4271.04	0.051	0.959	30.17	592.96

### Analysis of Sex Differences in Ventricular Volume

To compare whether there were differences in lateral ventricle volume, third ventricle volume, fourth ventricle volume, and ventricular system volume between males and females, independent samples *t*-tests were performed on the ventricle volume measurements measured by different methods, as shown in Table 3.The results showed that there was a significant difference in lateral ventricle volume (*P* < 0.01), third ventricle volume (*P* < 0.01), fourth ventricle volume (*P* < 0.05), and ventricular system volume (*P* < 0.01) that were significantly different between males and females, and lateral ventricle volume, third ventricle volume, fourth ventricle volume, and ventricular system volume were significantly higher in males than in females.

**Table 3:** Analysis of differences in ventricular volumes by gender as measured by ImageJ, 3D Slicer, FreeSurfer, and ITK-SNAP ($\overline{X }$±S)

Tool	Volume	Male (*n* = 39)	Female (*n* = 71)	*t*	*P*-value
ImageJ	LVV (mm^3^)	16,274.91 ± 4059.78	13,588.46 ± 3140.33	3.860	< 0.001
	TVV (mm^3^)	1270.43 ± 291.27	990.40 ± 238.93	5.434	< 0.001
	FVV (mm^3^)	1788.18 ± 387.92	1586.26 ± 407.38	2.528	0.013
	VV (mm^3^)	19,333.51 ± 4268.73	16,165.13 ± 3332.55	4.309	< 0.001
3D Slicer	LVV (mm^3^)	17,174.98 ± 4589.58	13,628.95 ± 3673.68	4.426	< 0.001
	TVV (mm^3^)	1252.54 ± 293.23	962.22 ± 226.17	5.368	< 0.001
	FVV (mm^3^)	1783.66 ± 389.31	1582.10 ± 416.54	2.484	0.015
	VV (mm^3^)	20,211.17 ± 4800.27	16,173.27 ± 3861.41	4.806	< 0.001
FreeSurfer	LVV (mm3)	14,891.78 ± 4319.42	12,091.55 ± 3529.09	3.672	< 0.001
	TVV (mm3)	1236.70 ± 232.66	952.23 ± 211.30	6.516	< 0.001
	FVV (mm3)	1778.00 ± 383.56	1590.73 ± 381.39	2.459	0.016
	VV (mm3)	17,906.48 ± 4456.14	14,634.50 ± 3705.54	4.119	< 0.001
ITK-SNAP	LVV (mm3)	15,851.06 ± 4395.26	12,695.07 ± 3306.36	4.250	< 0.001
	TVV (mm3)	1229.87 ± 297.89	965.78 ± 232.56	4.792	< 0.001
	FVV (mm3)	1794.43 ± 392.34	1585.33 ± 409.89	2.598	0.011
	VV (mm3)	18,875.36 ± 4623.23	15,246.19 ± 3473.55	4.649	< 0.001

Volume values in the table are expressed as mean ± standard deviation ($\overline{X }$±S) in mm.^3^

### Analysis of the Efficacy of Three Automated Measurement Methods to Assess Ventricular Volume

#### Correlation Analysis

The Pearson correlation coefficients between the results of the three automatic measurements and manual measurements of the volumes of each ventricle by 3D Slicer, FreeSurfer, and ITK-SNAP are shown in Table 4, from which it can be learned that the correlation coefficients between 3D Slicer and manual measurements in the lateral ventricle, third ventricle, fourth ventricle, and the ventricular systemic volume are 0.900, 0.972, and 0.995, respectively; 0.913, which were significantly correlated (*P* < 0.01), with the highest correlation coefficient for measurements of the fourth ventricle; the correlation coefficients between FreeSurfer and manual measurements on the volumes of the lateral ventricle, third ventricle, fourth ventricle, and ventricular system were 0.842, 0.938, 0.986, 0.861, respectively, with the highest correlation coefficients for measurements of the fourth ventricle (*P* < 0.01). The correlation coefficient was the highest (*P* < 0.01); the correlation coefficients between ITK-SNAP and manual measurements on the lateral ventricle, third ventricle, fourth ventricle, and ventricular system volumes were 0.929, 0.979, 0.999, and 0.939, respectively, which were significantly correlated (*P* < 0.01), with the highest correlation coefficients for the fourth ventricle measurements. In summary, 3D Slicer, ITK-SNAP, and FreeSurfer were significantly correlated with manual measurements on ventricular volume measurements, with 3D Slicer and ITK-SNAP having higher correlations with manual measurements, whereas FreeSurfer had a relatively low correlation with manual measurements, and all three had the highest correlation coefficients for fourth ventricle measurements.

**Table 4:** Pearson’s correlation analysis between automated and manual measurements of ventricular volume

	Lateral ventricles	Third ventricle	Fourth ventricle	Ventricles
3D Slicer	0.900^**^ (0.857 ~ 0.930)	0.972^**^ (0.959 ~ 0.981)	0.995^**^ (0.993 ~ 0.997)	0.913^**^ (0.875 ~ 0.940)
FreeSurfer	0.842^**^ (0.777 ~ 0.889)	0.938^**^ (0.911 ~ 0.957)	0.986^**^ (0.979 ~ 0.990)	0.861^**^ (0.803 ~ 0.902)
ITK-SNAP	0.929^**^ (0.898 ~ 0.951)	0.979^**^ (0.969 ~ 0.986)	0.999^**^ (0.998 ~ 0.999)	0.939^**^ (0.912 ~ 0.958)

Pearson correlation coefficients are shown in the table, ** indicates *P* < 0.01

The Pearson correlation coefficients between the 3D Slicer, FreeSurfer, and ITK-SNAP automated measurements and the manual measurements of each ventricular volume for both males and females are shown in Table 5. The correlation between the FreeSurfer and the manual measurements for the lateral and third ventricle volumes for females was strong (*r* = 0.777), and the remaining automated and manual measurements were all very strongly correlated (*r* > 0.8). Measurements were very strongly correlated with manual measurements (*r* > 0.8). For each ventricular volume measurement in males, the Pearson correlation coefficient between ITK-SNAP and manual measurements was the highest, followed by 3D Slicer, and FreeSurfer was the lowest. The Pearson correlation coefficient between ITK-SNAP and manual measurements was the highest, 3D Slicer was the next highest, and FreeSurfer was the lowest for each ventricular volume measurement in females.

**Table 5:** Pearson’s correlation analysis of ventricular volumes by sex measured by automated versus manual measurement methods

	Automatic measuring tools	Male	Female
Lateral ventricles	3D Slicer	0.916^**^ (0.845 ~ 0.956)	0.858^**^ (0.781 ~ 0.909)
	FreeSurfer	0.873^**^ (0.769 ~ 0.932)	0.777^**^ (0.664 ~ 0.855)
	ITK-SNAP	0.972^**^ (0.947 ~ 0.985)	0.870^**^ (0.799 ~ 0.917)
Third ventricle	3D Slicer	0.945^**^ (0.897 ~ 0.971)	0.961^**^ (0.886 ~ 0.967)
	FreeSurfer	0.873^**^ (0.856 ~ 0.897)	0.777^**^ (0.654 ~ 0.858)
	ITK-SNAP	0.972^**^ (0.945 ~ 0.985)	0.970^**^ (0.963 ~ 0.985)
Fourth ventricle	3D Slicer	0.994^**^ (0.988 ~ 0.997)	0.995^**^ (0.993 ~ 0.997)
	FreeSurfer	0.989^**^ (0.979 ~ 0.994)	0.983^**^ (0.973 ~ 0.990)
	ITK-SNAP	0.998^**^ (0.997 ~ 0.999)	0.999^**^ (0.998 ~ 0.999)
Ventricles	3D Slicer	0.926^**^ (0.862 ~ 0.961)	0.872^**^ (0.802 ~ 0.919)
	FreeSurfer	0.883^**^ (0.786 ~ 0.937)	0.801^**^ (0.698 ~ 0.871)
	ITK-SNAP	0.975^**^ (0.953 ~ 0.987)	0.884^**^ (0.820 ~ 0.926)

Pearson correlation coefficients are shown in the table, ** indicates *P* < 0.01

Furthermore, we compared the correlation among different methods; 3D Slicer and ITK-SNAP show the best correlations (*r* > 0.90) for all methods we compared (Table S1).

#### Reliability Analysis

ICC was used to assess the reliability of 3D Slicer, FreeSurfer, and ITK-SNAP measurements of ventricular volumes, and the intragroup correlation coefficients ICC and 95% confidence intervals for the automated measurements versus the manual methods, respectively, are shown in Table 6. For lateral ventricle volume, 3D Slicer, FreeSurfer, and ITK-SNAP measurements were in good agreement with those using manual measurements, with ICC values greater than 0.8, with ITK-SNAP having the highest ICC value (ICC = 0.927), 3D Slicer having the second highest ICC value (ICC = 0.889), and FreeSurfer having the lowest ICC value (ICC = 0.839). For third ventricle volume, 3D Slicer, FreeSurfer, and ITK-SNAP measurements were in excellent agreement with those using manual measurements, with ICC values greater than 0.9, with ITK-SNAP having the highest ICC value (ICC = 0.979), 3D Slicer having the second highest ICC value (ICC = 0.972), and FreeSurfer having the lowest ICC value (ICC = 0.931). For fourth ventricle volume, 3D Slicer, FreeSurfer, and ITK-SNAP measurements showed excellent agreement with those using manual measurements, with ICC values greater than 0.9, with ITK-SNAP having the highest ICC value (ICC = 0.999), 3D Slicer having the second highest ICC value (ICC = 0.995), and FreeSurfer having the lowest ICC value (ICC = 0.985). For ventricular system volumes, 3D Slicer, FreeSurfer, and ITK-SNAP measurements were in good agreement with those using manual measurements, with ICC values greater than 0.8, with ITK-SNAP having the highest ICC value (ICC = 0.936), 3D Slicer having the second highest ICC value (ICC = 0.903), and FreeSurfer having the lowest ICC value (ICC = 0.858). In summary, for the measurement of each ventricular volume, 3D Slicer, FreeSurfer, and ITK-SNAP had good agreement with the manual measurement, which was greater than 0.8, with the three automated measurements having the highest ICC values for measuring the volume of the fourth ventricle (0.995–0.999), and measuring the volume of the lateral ventricle (0.839–0.927) being the lowest.

**Table 6:** Intra-group correlation coefficients (ICC) and 95% confidence intervals for automated measurements, respectively, with manual measurement methods

	3D Slicer	FreeSurfer	ITK-SNAP
Lateral ventricles	0.889^**^ (0.842 ~ 0.922)	0.839^**^ (0.773 ~ 0.886)	0.927^**^ (0.895 ~ 0.949)
Third ventricle	0.972^**^ (0.959 ~ 0.981)	0.931^**^ (0.901 ~ 0.952)	0.979^**^ (0.969 ~ 0.985)
Fourth ventricle	0.995^**^ (0.993 ~ 0.997)	0.985^**^ (0.977 ~ 0.989)	0.999^**^ (0.998 ~ 0.999)
Ventricles	0.903^**^ (0.861 ~ 0.932)	0.858^**^ (0.800 ~ 0.901)	0.936^**^ (0.908 ~ 0.956)

** in the table indicates *P* < 0.01

The ICCs and 95% confidence intervals of 3D Slicer, FreeSurfer, and ITK-SNAP for the measurement of each ventricular volume in males and females, and the ICCs and 95% confidence intervals for the measurement of each ventricular volume in males and females between the three automated measurements and manual measurements for the different genders, are shown in Table 7. For males, 3D Slicer, FreeSurfer, and ITK-SNAP showed good agreement with manual measurements, all of which were greater than 0.8, with ITK-SNAP having the highest ICC with manual measurements, 3D Slicer the next highest, and FreeSurfer the lowest. For the measurement of the volume of each ventricle in females, 3D Slicer, FreeSurfer, and ITK-SNAP showed good agreement with manual measurements, all greater than 0.7. The ICC values between ITK-SNAP and manual measurements were the highest, 3D Slicer the next highest, and FreeSurfer the lowest.

**Table 7:** ICC and 95% confidence intervals of ventricular volumes of different genders measured by automated versus manual methods, respectively

	Automatic measuring tools	Male	Female
Lateral ventricles	3D Slicer	0.910^**^ (0.834 ~ 0.952)	0.848^**^ (0.766 ~ 0.902)
	FreeSurfer	0.871^**^ (0.768 ~ 0.930)	0.772^**^ (0.657 ~ 0.851)
	ITK-SNAP	0.969^**^ (0.941 ~ 0.984)	0.869^**^ (0.798 ~ 0.916)
Third ventricle	3D Slicer	0.945^**^ (0.898 ~ 0.971)	0.976^**^ (0.962 ~ 0.985)
	FreeSurfer	0.882^**^ (0.786 ~ 0.936)	0.929 ^**^ (0.889 ~ 0.955)
	ITK-SNAP	0.971^**^ (0.945 ~ 0.985)	0.980^**^ (0.968 ~ 0.987)
Fourth ventricle	3D Slicer	0.994^**^ (0.988 ~ 0.997)	0.995^**^ (0.992 ~ 0.997)
	FreeSurfer	0.989^**^ (0.979 ~ 0.994)	0.981^**^ (0.970 ~ 0.988)
	ITK-SNAP	0.998^**^ (0.997 ~ 0.999)	0.999^**^ (0.998 ~ 0.999)
Ventricles	3D Slicer	0.919^**^ (0.852 ~ 0.957)	0.863^**^ (0.789 ~ 0.912)
	FreeSurfer	0.882^**^ (0.786 ~ 0.936)	0.796^**^ (0.692 ~ 0.868)
	ITK-SNAP	0.972^**^ (0.948 ~ 0.985)	0.883^**^ (0.819 ~ 0.925)

** in the table indicates *P* < 0.01

#### Coherence Analysis

**Fig. 7:**
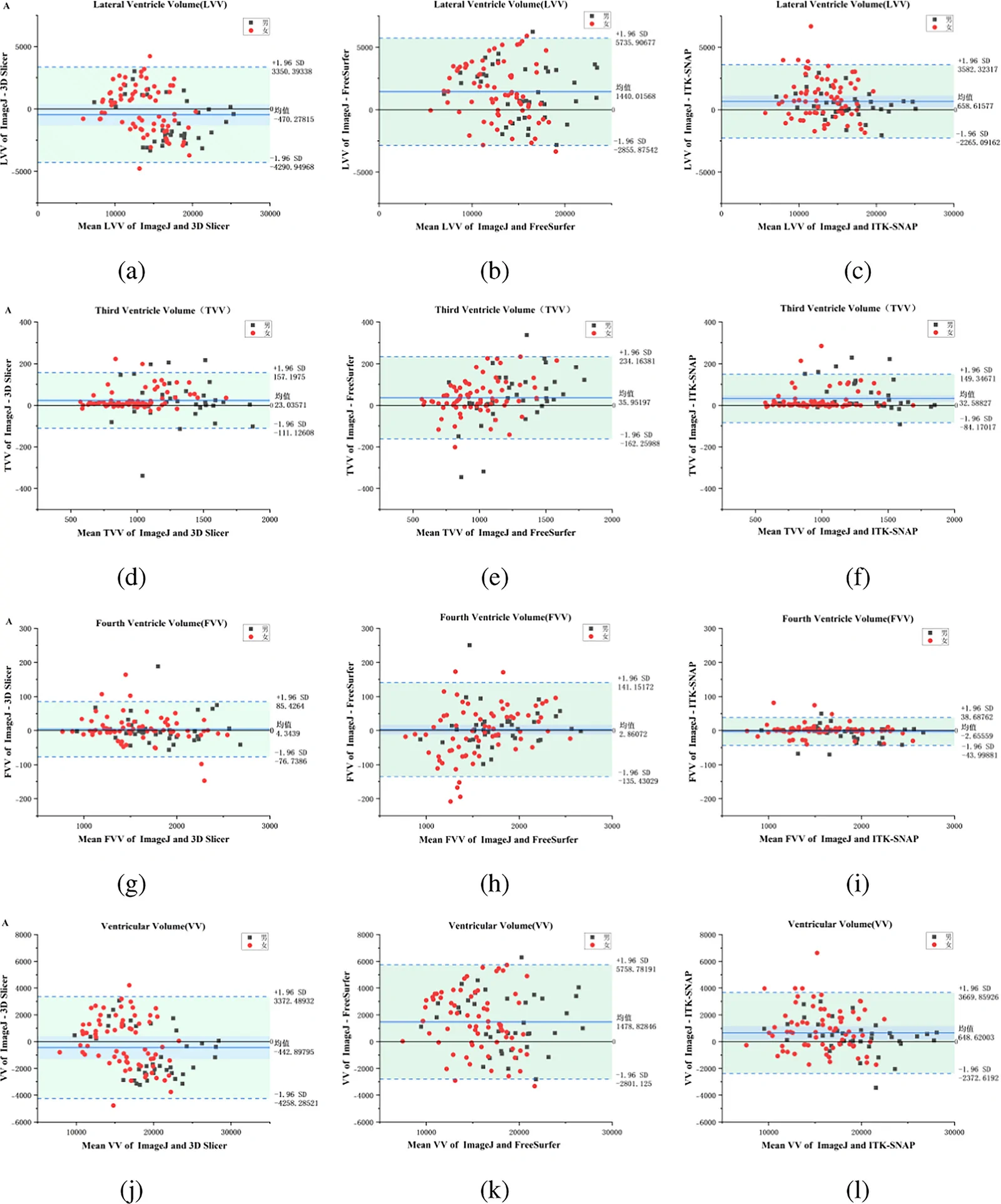
The Bland–Altman plots of each ventricular volume for manual and three automated measurement methods. **a**–**c** The Bland–Altman plots of ImageJ versus 3D Slicer, FreeSurfer, and ITK-SNAP lateral ventricle volumes, respectively; **d**–**f** The Bland–Altman plots of ImageJ versus 3D Slicer, FreeSurfer, and ITK-SNAP third ventricle volumes, respectively. Altman plots; **g**–**i** The Bland–Altman plots of ImageJ versus 3D Slicer, FreeSurfer, and ITK-SNAP fourth ventricle volumes, respectively; **j**–**l** ImageJ versus 3D Slicer, FreeSurfer, and ITK-SNAP ventricular system volumes, respectively. In the Bland–Altman plots the *x*-axis represents the mean of the two software measurements and the *y*-axis represents the difference between the two software measurements. The units for both the *x*- and *y*-axes are mm^3^. The red circle and black square indicate female and male, respectively. The blue horizontal dashed lines delineate the 95% confidence intervals (the likelihood of individual measures to be within ± 1.96 SDs). The black horizontal line represents the equal (the difference between two software measurements is zero) line. The blue horizontal line indicates the difference between two software measurements

The results of the Bland–Altman analysis in Fig. 7 show that for the measurement of lateral ventricle volume, the 95% limit of agreement (LoA) between the 3D Slicer measurement of lateral ventricle volume results and the manual measurement of lateral ventricle volume results was − 470.28 ± 3820.67, the FreeSurfer measurement of lateral ventricle volume results and the manual measurement of lateral ventricle volume results had a LoA of 1440.02 ± 4295.89, and the LoA between the results of lateral ventricular volume measurement by ITK-SNAP and the results of manual measurement of lateral ventricular volume was 658.62 ± 2923.71. The 95% limits of agreement (LoA) were FreeSurfer, 3D Slicer, and ITK-SNAP in descending order of broadness, indicating that ITK-SNAP showed higher agreement with manual measurements than FreeSurfer and 3D Slicer. The 95% differences of the lateral ventricle volume measurements were all within the 95% consistency boundaries, and the scatter distributions of 3D Slicer and ITK-SNAP were more concentrated, indicating that the agreement between 3D Slicer and ITK-SNAP and manual measurements was better. In contrast, FreeSurfer’s measurement results have a large fluctuation in the spread, indicating that the consistency of its measurement results is relatively poor.

For the third ventricle volume measurements, the LoA between the 3D Slicer third ventricle volume results and the manual third ventricle volume results was 23.04 ± 134.16, the LoA between the FreeSurfer third ventricle volume results and the manual lateral ventricle volume results was 35.95 ± 198.21, and the LoA between the ITK-SNAP third ventricle volume results and the manual measurement of the third ventricle volume results was 32.59 ± 116.76. The 95% LoA were FreeSurfer, 3D Slicer, and ITK-SNAP in descending order of broadness, suggesting that the ITK-SNAP showed higher agreement with the manual measurement results than FreeSurfer and 3D Slicer. The scatters of 3D Slicer and ITK-SNAP ventricular volume measurements were mainly concentrated within the 95% consistency boundaries, and most of the scatters were located near the difference of 0 boundaries, suggesting that the agreement between 3D Slicer and ITK-SNAP and manual measurements was better. Many of the scatters of FreeSurfer’s measurements were located near the difference of 0 boundaries, but the differences fluctuated more, indicating relatively poor agreement between its measurements and the manual measurements.

For the fourth ventricle volume measurements, the LoA between the 3D Slicer and manual fourth ventricle volume results was 4.34 ± 81.08, the LoA between the FreeSurfer and manual fourth ventricle volume results was 2.86 ± 138.29, and the LoA between the ITK-SNAP and manual fourth ventricle volume results was − 2.66 ± 41.34. The 95% LoA were, in descending order, − 2.66 ± 41.34 between the FreeSurfer, 3D Slicer, and 3D-SNAP fourth ventricle volume results. The LoA between the results and the manual measurement of fourth ventricle volume results was − 2.66 ± 41.34. The 95% LoA were FreeSurfer, 3D Slicer, and ITK-SNAP in descending order of broadness, indicating that the agreement between ITK-SNAP and the manual measurement results was higher than that of FreeSurfer and 3D Slicer. 3D Slicer’s fourth ventricle volume measurements had differences mainly concentrated within the 95% consistency boundaries, indicating better agreement between 3D Slicer and manual measurements; FreeSurfer’s measurements had more scatter located within the 95% consistency boundaries’ scatter, but there were also more scatter distributions outside the boundaries, with larger fluctuations in the differences, indicating relatively poorer consistency of its measurements; the difference of the measurement results of ITK-SNAP has some scatters located outside the 95% consistency boundaries, but most of the scatters are mainly concentrated within the 95% consistency boundaries, with the narrowest LoA, which indicates that the consistency between ITK-SNAP and the manual measurement results is higher than that of 3D Slicer and FreeSurfer.

For the ventricular system volume measurements, the LoA between the 3D Slicer and manual ventricular system volume results was − 442.90 ± 3815.39, the LoA between the FreeSurfer and manual ventricular system volume results was 1478.83 ± 4279.95, and the LoA between the ITK-SNAP and manual ventricular system volume results was 648.62 ± 3021.24. The 95% limits of agreement (LoA) were, in descending order, broader to narrower for the FreeSurfer and ITK-SNAP. The LoA between the results of measuring the ventricular system volume and the results of manually measuring the ventricular system volume was 648.62 ± 3021.24. The 95% limits of agreement (LoA) were in order from broadest to narrowest for FreeSurfer, 3D Slicer, and ITK-SNAP, suggesting that the agreement between ITK-SNAP and the results of the manual measurements was higher than that for FreeSurfer and 3D. The differences of the 3D Slicer’s ventricular system volume measurements were almost entirely concentrated within the 95% consistency limits, indicating that the agreement between the 3D Slicer and the manual measurements was better; the differences of the FreeSurfer’s measurements were mainly concentrated within the 95% consistency limits, and the differences fluctuated greatly, indicating that the consistency of its measurements was relatively poor; and the differences of the ITK-SNAP’s measurement results were mainly concentrated within the 95% consistency limit, and the LoA limit was the narrowest, with smaller fluctuation of the difference.

**Fig. 8:**
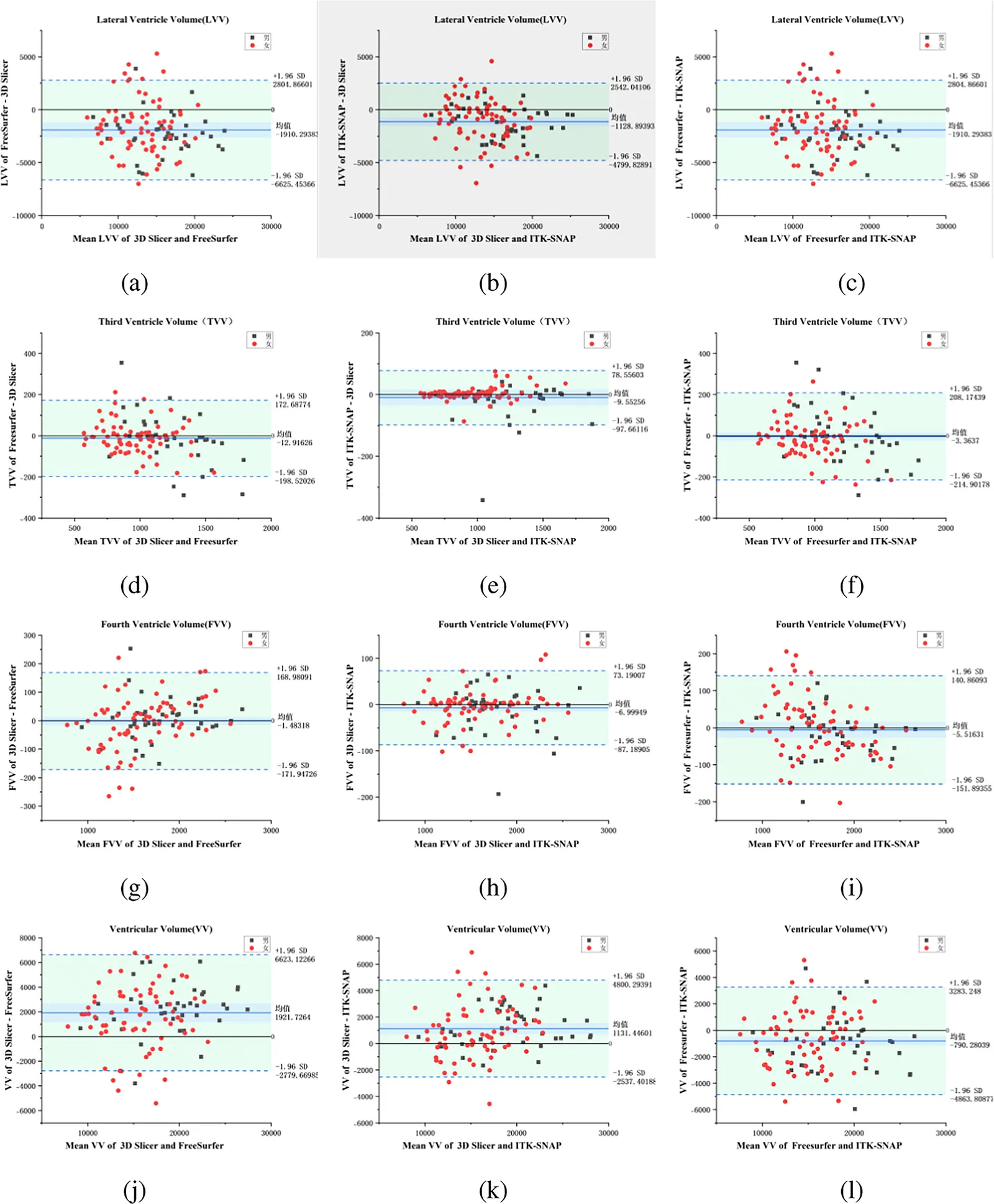
The Bland–Altman plots comparing ventricular volumes between 3D Slicer and FreeSurfer, 3D Slicer and ITK-SNAP, and FreeSurfer and ITK-SNAP. **a**–**c** Lateral ventricle volumes; **d**–**f** third ventricle volumes; **g**–**i** fourth ventricle volumes; **j**–**l** total ventricular system volumes

The results of the Bland–Altman analysis in Fig. 8 show the measurement of ventricle volumes between 3D Slicer and FreeSurfer, 3D Slicer and ITK-SNAP, and ITK-SNAP and FreeSurfer. For the LVV, TVV, FVV, and VV measurements, 3D Slicer and ITK-SNAP show the best agreement for all methods we compared (Fig. 8b, e, h, k).

In each plot, the *x*-axis represents the mean of the two software measurements, and the *y*-axis represents the difference between them. Both axes use units of mm^3^. Red circles denote female participants, and black squares denote male participants. The blue horizontal dashed lines indicate the 95% limits of agreement (± 1.96 SD). The solid black horizontal line represents zero difference, and the solid blue horizontal line represents the mean difference between the two software measurements.

### Three-Dimensional Visualization of Segmented Ventricles with Three Automated Measurement Tools

**Fig. 9:**
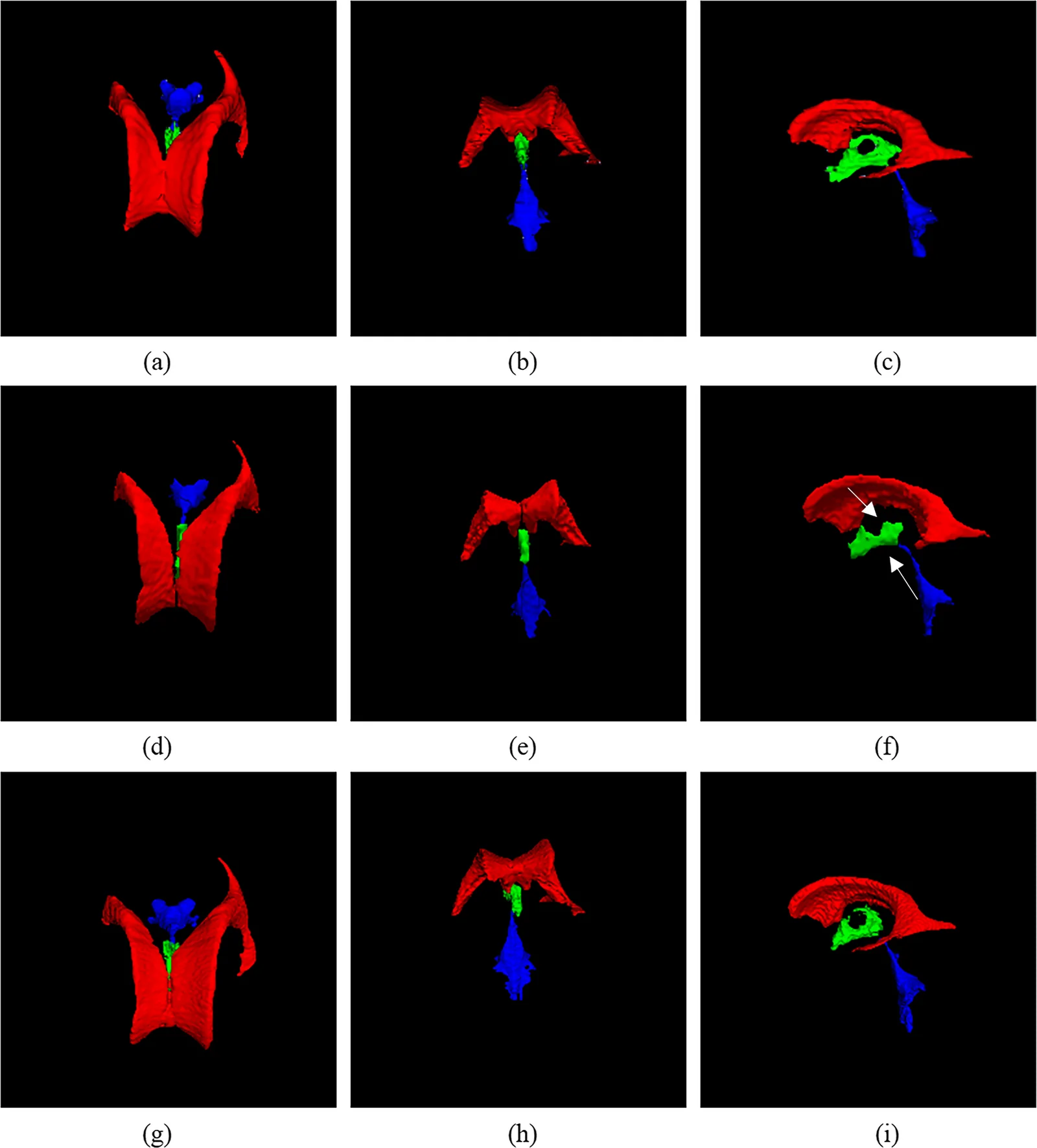
3D visualization of three automated measurements of segmented ventricles. Red indicates lateral ventricle, green indicates third ventricle, blue indicates fourth ventricle, and arrows indicate incomplete segmentation. **a**–**c** Visualizations of 3D Slicer axial, coronal, and sagittal positions, **d**–**f** visualizations of FreeSurfer axial, coronal, and sagittal positions, and **g**–**i** visualizations of ITK-SNAP axial, coronal, and sagittal positions, respectively

In order to observe the effect of ventricular segmentation by the three automated measurement tools, three-dimensional reconstruction of the automated measurement segmentation results of one subject was visualized in axial, coronal, and sagittal planes. As shown in Fig. 9, the lateral ventricles are shown in red, the third ventricle in green, and the fourth ventricle in blue. The visualization results showed that FreeSurfer segmented incompletely the lower horn of the lateral ventricle and the top of the third ventricle, and completely segmented the fourth ventricle; 3D Slicer segmented completely the lateral ventricle, the third ventricle, and the fourth ventricle; and ITK-SNAP segmented completely the lateral ventricle, the third ventricle, and the fourth ventricle.

### Comparison of the Main Features of Three Automated Measurement Tools

In order to analyze the efficacy of the three automated measurement tools in terms of practicality, several of their main features are compared in Table 8.

**Table 8:** Comparison of key features of 3D Slicer, ITK-SNAP, and FreeSurfer

	3D Slicer	ITK-SNAP	FreeSurfer
Internet address	https://www.slicer org	http://www.itksnap.org	https://surfer.nmr.mgh.harvard.edu
Requires download and installation	Yes	Yes	Yes
OS platform	Linux, Mac OS, Microsoft Windows	Linux, Mac OS, Microsoft Windows	Linux, Mac OS
Input image modalities	DICOM	DICOM and NIFTI	DICOM and NIFTI
Input file type	T1-MRI, DWI-MRI, CT, CTA	T1-MRI, T2-MRI, CT	T1-MRI
Need for interaction with human user	Parameter tuning and boundary refinement	Parameter tuning and boundary refinement	Fully automatic
Need for software add-ons	Yes	No	No
Processing speed	20 min	30 min	4 ~ 6 h
Brain anatomical structure labeling	Manual	Manual	Automatic
Need for program	No	No	Yes

*MRI* magnetic resonance imaging, *DWI* diffusion-weighted imaging, *CT* computed tomography, *CTA* computed tomography angiography, *NIFTI* Neuroimaging Informatics Technology Initiative, *DICOM* digital imaging and communication in medicine

In terms of segmentation efficiency, average processing time was ~ 90 min per subject for manual segmentation. However, for users skilled in automated measurement tools, it takes an average of 20 min to complete the volume measurement of the ventricular system of one subject using 3D Slicer [45], an average of 30 min for ITK-SNAP measurement [44], and an average of 4 ~ 6 h to segment all brain anatomical structures using FreeSurfer [45]. It does not require human intervention and relies entirely on automatic segmentation, which can be run automatically by the user by simply typing commands and can complete the automatic segmentation of all brain anatomical structures. In addition, it can also automatically segment a certain brain anatomical structure individually, thus shortening the segmentation time.

In terms of user interaction experience, 3D Slicer’s advantage is that in addition to automatic segmentation of brain structures, it can also perform manual correction at the same time [33], and ITK-SNAP can also be manually modified after automatic segmentation, both of which provide easy-to-operate software interfaces and can also be calibrated on three-dimensional images, which not only improves the efficiency but also improves the accuracy. FreeSurfer, on the other hand, needs to input commands to modify it only after the segmentation is completed, and it is impossible to see the target segmented brain structure directly, which brings great difficulties to users. In addition, ITK-SNAP provides video tutorials on its official website, and the software is easy to use and has a short learning curve so that even users without a deep mathematical background are able to carry out complex image segmentation tasks. 3D Slicer provides full functionality but has a long learning curve, while FreeSurfer requires some basic programming knowledge.

## Discussion

The results from the four measurement tools (ImageJ, 3D Slicer, FreeSurfer, ITK-SNAP) showed no statistically significant differences between the raw data and the standardized data (*P* > 0.05), indicating that the standardization process effectively eliminates individual variability and provides a reliable foundation for subsequent analysis. This finding is consistent with previous research highlighting the importance of the 3D Slicer standardization process in measuring ventricular volume [33]. On the other hand, all measurement methods revealed significantly higher ventricular volume metrics in males compared to females (LVV, TVV, FVV, VV; all *P* < 0.05). Similar results reported before, men exhibited larger average ventricular volumes than women, and individuals in their 40 s had greater volumes than those in their 20 s [13, 46]. Age-related ventricular enlargement was more pronounced in men. Additionally, ventricular volume correlated positively with body height in men, but no such association was observed in women [47]. This sex-specific disparity may relate to anatomical variations in physiological structures, though further validation incorporating cerebrospinal fluid dynamics or hormonal influences is required. 3D Slicer exhibited the highest correlation (fourth ventricle: *r* = 0.995), likely due to its algorithm’s suitability for reconstructing complex curved structures. This precision mirrors the advantages of surface-reconstruction algorithms in 3D Slicer. FreeSurfer showed the lowest correlation in lateral ventricle measurements (*r* = 0.832), which may be due to the complexity of the lateral ventricle structure and limitations in identifying the boundaries of small structures, though its open-source nature retains clinical utility. ITK-SNAP demonstrated balanced performance (all ventricles: *r* > 0.870), combining accuracy with multimodal compatibility, paralleling developments in machine learning-assisted 3D Slicer analysis. The high consistency between automated tools and the gold standard (ImageJ manual measurements; *r* > 0.800) supports their clinical feasibility as replacements for traditional manual methods. Precision variations across tools suggest scenario-based selections: prioritize ITK-SNAP for high accuracy and 3D Slicer for efficiency in screening. Current automated tools show relatively higher errors in measuring small-volume structures like the third ventricle (minimum *r* = 0.842), necessitating optimized algorithms for small-scale anatomical recognition. Similar challenges arose in 3D Slicer development, where deep learning-enhanced edge detection offers potential solutions. Additionally, we note that differences under 5% between ITK-SNAP and manual segmentation fall below the typical diagnostic thresholds used for assessing ventricular enlargement in hydrocephalus (Evans’ Index ≥ 0.3). This suggests that both ITK-SNAP and 3D Slicer provide clinically acceptable accuracy for diagnostic support. Although the automated tools evaluated in this study showed high correlations with manual measurements, measurement error must still be considered in clinical contexts. Minor volumetric discrepancies are unlikely to alter routine clinical decisions in hydrocephalus or dementia [48], whereas larger, systematic deviations may reduce sensitivity for detecting early atrophic changes and therefore influence longitudinal or early-stage diagnoses [49–51].

This study has several limitations. The sample size is relatively small, and no a priori power calculation was performed, which represents a methodological limitation. As a pilot study conducted in a demographically homogeneous cohort, our findings establish a normative performance baseline but require further validation in other ethnic groups, older populations, and disease-specific cohorts. In addition, we did not compare the efficacy of the examined tools with several newer AI-based segmentation platforms, such as volBrain, ALVIN, MRICloud, NeuroQuant, and SynthSeg. volBrain is a web-based MRI volumetry pipeline that provides automated brain volume measurements through a simple web interface (http://volbrain.upv.es) [52]. Automatic Lateral Ventricle Delineation (ALVIN) is a fully automated, segmentation-based algorithm that provides lateral ventricular volumes normalized to head size, reporting the combined volume of both ventricles [53]. MRICloud is an online platform capable of automatically segmenting structural MRI into 287 labeled brain regions [54]. NeuroQuant (NQ), the first FDA-approved volumetric analysis software, is a standalone, fully automated pipeline in which the brain is inflated to a spherical shape, mapped to a common spherical space using Talairach coordinates, segmented, and then deflated back to its original shape. Regional brain volumes are normalized to intracranial volume and expressed as percentages [55]. SynthSeg is based on the SynthSeg framework, which uses synthesized training images to achieve high generalizability; it requires only labeled images for training and represents the first accurate method for automatic ultrahigh-resolution segmentation. SynthSeg is robust to variations in contrast and resolution and is available at https://github.com/chiara-mauri/claustrum_segmentation as well as within the FreeSurfer neuroimaging toolkit [56].

### Conclusions and Future Work

In this study, the ventriculometry volume results of 3D Slicer, FreeSurfer, and ITK-SNAP were comprehensively analyzed, and the correlation was ITK-SNAP, 3D Slicer, and FreeSurfer in descending order; the reliability was ITK-SNAP, 3D Slicer, and FreeSurfer in descending order; and the consistency was ITK-SNAP, 3D Slicer, and FreeSurfer in descending order. ITK-SNAP, 3D Slicer, FreeSurfer; reliability from high to low; consistency from high to low. 3D Slicer, FreeSurfer, and ITK-SNAP have high consistency and reliability with the manual measurement of ImageJ ventricular volume values, and 3D Slicer, FreeSurfer, and ITK-SNAP can be used as automated tools for ventricular volume measurement. However, in terms of overall performance, ITK-SNAP was the best for ventricular volume measurement, followed by 3D Slicer and FreeSurfer, and ITK-SNAP was the better automated measurement method in terms of reliability and consistency, which can be promoted for use by researchers and clinicians.

This study used a healthy population to examine differences in the efficacy of automated measurement tools, providing a benchmark for clinical research, and further validation and testing of the methods in patient populations with pathological characteristics will be conducted in the future. In addition, only three commonly used automated measurement methods were compared in this study, and new methods will continue to be added to compare their accuracy and reliability to promote the popularity and application of automated measurement methods in various fields. Emerging AI-based segmentation platforms such as volBrain, ALVIN, MRICloud, NeuroQuant, and SynthSeg demonstrate promising performance. Future comparative studies incorporating these systems are warranted to expand clinical applicability. By comparing three automated measurement methods, this study found that by combining back-end algorithms with a front-end interface, image segmentation and volume measurement tasks can be easily accomplished even if the user is not familiar with the complex algorithms. It is believed that a multimodal automated measurement tool integrating multiple algorithms can be developed in the future, which seamlessly combines advanced algorithms on the back-end with an intuitive and easy-to-use front-end user interface. This platform will provide powerful features, including support for multiple imaging modalities such as MRI, CT, and PET, an easy-to-operate platform interface, and the ability to flexibly apply different automated measurement algorithms, making it possible for even users without a deep mathematical background to carry out complex image segmentation tasks, and to complete high-precision measurements and analyses. Through this innovative tool, it provides more comprehensive decision-making for clinical diagnosis and treatment and ultimately achieves the goals of personalized medicine and precision treatment.

## Supplementary Information

Below is the link to the electronic supplementary material.

ESM1 (DOCX 12.3 KB)

## Data Availability

Data may be requested by email from the corresponding author.

## Code Availability

Any access and transfers of data must, however, fulfill necessary requirements such as local regulatory, ethics, and law.
