# Supplementary material for: Comparison of Three Automated Measurements of Ventricular Volumes of the Brain
Source: J Imaging Inform Med. 2026 Jan 20;39(5):4355–76. doi: 10.1007/s10278-025-01819-6 (PMC13694576; doi:10.1007/s10278-025-01819-6)
Supplement: Supplementary file 1 [file 10278_2025_1819_MOESM1_ESM.docx]

Table S1

|  | Lateral ventricles | Third ventricle | Fourth ventricle | ventricles |
| --- | --- | --- | --- | --- |
| 3D Slicer and ITK-SNAP | 0.903^**^(0.861~0.932) | 0.989^**^(0.984~0.992) | 0.995^**^(0.993~0.997) | 0.915^**^(0.878~0.941) |
| 3D Slicer and FreeSurfer | 0.841^**^(0.775~0.888) | 0.945^**^(0.920~0.962) | 0.979^**^(0.969~0.985) | 0.860^**^(0.802~0.902) |
| ITK-SNAP and FreeSurfer | 0.867^**^(0.811~0.907) | 0.926^**^(0.894~0.949) | 0.985^**^(0.978~0.989) | 0.881^**^(0.831~0.917) |
